# Computational Modelling of Metastasis Development in Renal Cell Carcinoma

**DOI:** 10.1371/journal.pcbi.1004626

**Published:** 2015-11-23

**Authors:** Etienne Baratchart, Sébastien Benzekry, Andreas Bikfalvi, Thierry Colin, Lindsay S. Cooley, Raphäel Pineau, Emeline J Ribot, Olivier Saut, Wilfried Souleyreau

**Affiliations:** 1 INRIA Bordeaux-Sud-Ouest team MONC; University of Bordeaux, IMB, UMR 5251; CNRS, IMB, UMR 5251; Bordeaux INP, IMB, UMR 5251,Talence, France; 2 LAMC, INSERM U1029, University of Bordeaux, Pessac, France; 3 Centre de Résonance Magnétique des Systèmes Biologiques, UMR 5536, CNRS/University of Bordeaux Segalen, Bordeaux, France; University of California, Irvine, UNITED STATES

## Abstract

The biology of the metastatic colonization process remains a poorly understood phenomenon. To improve our knowledge of its dynamics, we conducted a modelling study based on multi-modal data from an orthotopic murine experimental system of metastatic renal cell carcinoma. The standard theory of metastatic colonization usually assumes that secondary tumours, once established at a distant site, grow independently from each other and from the primary tumour. Using a mathematical model that translates this assumption into equations, we challenged this theory against our data that included: 1) dynamics of primary tumour cells in the kidney and metastatic cells in the lungs, retrieved by green fluorescent protein tracking, and 2) magnetic resonance images (MRI) informing on the number and size of macroscopic lesions. Critically, when calibrated on the growth of the primary tumour and total metastatic burden, the predicted theoretical size distributions were not in agreement with the MRI observations. Moreover, tumour expansion only based on proliferation was not able to explain the volume increase of the metastatic lesions. These findings strongly suggested rejection of the standard theory, demonstrating that the time development of the size distribution of metastases could not be explained by independent growth of metastatic foci. This led us to investigate the effect of spatial interactions between merging metastatic tumours on the dynamics of the global metastatic burden. We derived a mathematical model of spatial tumour growth, confronted it with experimental data of single metastatic tumour growth, and used it to provide insights on the dynamics of multiple tumours growing in close vicinity. Together, our results have implications for theories of the metastatic process and suggest that global dynamics of metastasis development is dependent on spatial interactions between metastatic lesions.

## Author Summary

We used mathematical modelling to formalize the standard theory of metastatic initiation, under which secondary tumours, after establishment in a distant organ, grow independently from each other and from the primary tumour. When calibrated on the experimental data of primary tumour and total metastatic burden in the lungs in an animal model of renal cell carcinoma, the initial model predicted a size distribution of metastatic foci that did not fit with observations obtained experimentally using magnetic resonance imaging (which provided size and number of macro-metastases). The model predicted an increase in the number of lesions, but of smaller size when compared to the data. This led us to revise the standard theory and to propose two hypotheses in order to explain the observations: 1) small metastatic foci merge into larger ones and/or 2) circulating tumour cells may join already established tumours. We then derived a spatial model of tumour growth in order to explore the quantitative implications of tumours merging on global tumour growth and estimated the numbers of required metastatic foci to obtain the observed metastatic volumes.

## Introduction

Metastasis, the spread of cancer cells from a primary tumour to secondary location(s) in the body, is the ultimate cause of death for the majority of cancer patients [1,2]. Although studied for more than 180 years [3], increasing efforts in recent years contributed to a better understanding of this aspect of tumour development [2,4], with exciting new discoveries [5–8] that potentially have important clinical implications. The metastatic process can be coarsely divided into two major phases: 1) dissemination of detaching cells from the primary tumour to a secondary site and 2) colonization of this distant organ [1,9]. While the former has been relatively well elucidated, in particular due to recent advances about the epithelial-to-mesenchymal transition [10] and advances on our understanding of molecular and genetic determinants [11,12], the latter remains not fully understood, especially during the colonization phase [1,12]. This is due, in part, to experimental limitations that hinder our ability to observe colonization of organs by tumour cells and the development of tumour lesions.

In this context, mathematical models provide powerful tools to potentiate data analysis, infer hidden information, test biological hypotheses against the empirical data and simulate a range of conditions that may be confronted to the biological reality. In recent years, several models for tumour growth have been developed (see [13,14] for historical reviews), based on multiple modelling techniques from non-spatial ordinary differential equations models (see [15] for a benchmark of these against experimental *in vivo* data) to discrete agent-based models [16–18] and continuous partial differential equations based on tissue mechanics laws [19,20]. However, despite a large body of literature for modelling tumour growth, relatively little effort has been devoted to the development and validation of mathematical models describing the biology of the metastatic process (see [21,22] for an early and notable exception, [23,24] for more recent studies and [25] for a recent review). In 2000, Iwata and colleagues proposed a simple mathematical model for the growth of a population of metastatic colonies [26], which was recently shown able to fit experimental data describing the increase in total metastatic burden [27,28]. In this mathematical description, each metastasis grows independently from the others and from the primary tumour. We report herein a theoretical study to test this hypothesis using *in vivo* data derived from a metastatic renal carcinoma model in mice. We show that the standard theory of metastatic initiation in which distinct foci grow independently from each other (as assumed in [21]) predicted an unrealistically large number of metastases, while the tumours sizes were too small.

In a space-limited organ (such as the lungs), where two neighbouring metastatic foci are growing in close vicinity, they might enter in contact and interactions occur, ultimately leading to the merging of the metastatic foci. This phenomenon is not taken into account in a classical description of metastasis development, although it can lead to important differences in the number and sizes of the colonies. Moreover, mechanical interactions could occur during metastases merging, possibly impacting the global dynamics. Therefore, we next conducted a simulation study to quantify the effect of mechanical interactions between two neighbouring tumours. Based on mechanical laws for tissue growth, we derived a minimally parameterized model (2 parameters). This second, spatial model, based on a pressure-mediated growth law, once fitted to magnetic resonance imaging data of individual metastatic tumour growths, offered an adapted framework to perform simulations of spatially interacting tumours. These revealed significant impact of the interactions on the global growth and allowed to test if merging by passive motion could explain the data that are not in accordance with the classical model.

To our knowledge, this is the first time that data on size distribution of metastasis at this resolution (with such a small visibility threshold, of the order of 0.05 mm^3^) is reported and analysed in lights of a theoretical model.

## Results

### Data-driven modelling of metastatic growth

As an initial step, we studied the growth rates of individual metastatic tumours. Then, we calibrated a more elaborated mathematical model of tumour growth and metastatic dissemination using quantitative data derived from green fluorescent protein (GFP)-tracking of primary and metastatic tumours (see Materials and methods, *n =* 31 mice). Finally, we used the model to investigate predictions of the standard theory with regard to number and sizes of metastatic lesions and compared them to Magnetic Resonance Imaging (MRI) data (see Materials and methods, *n =* 6 mice).

#### Growth rates of individual metastatic tumours

**Fig 1:**
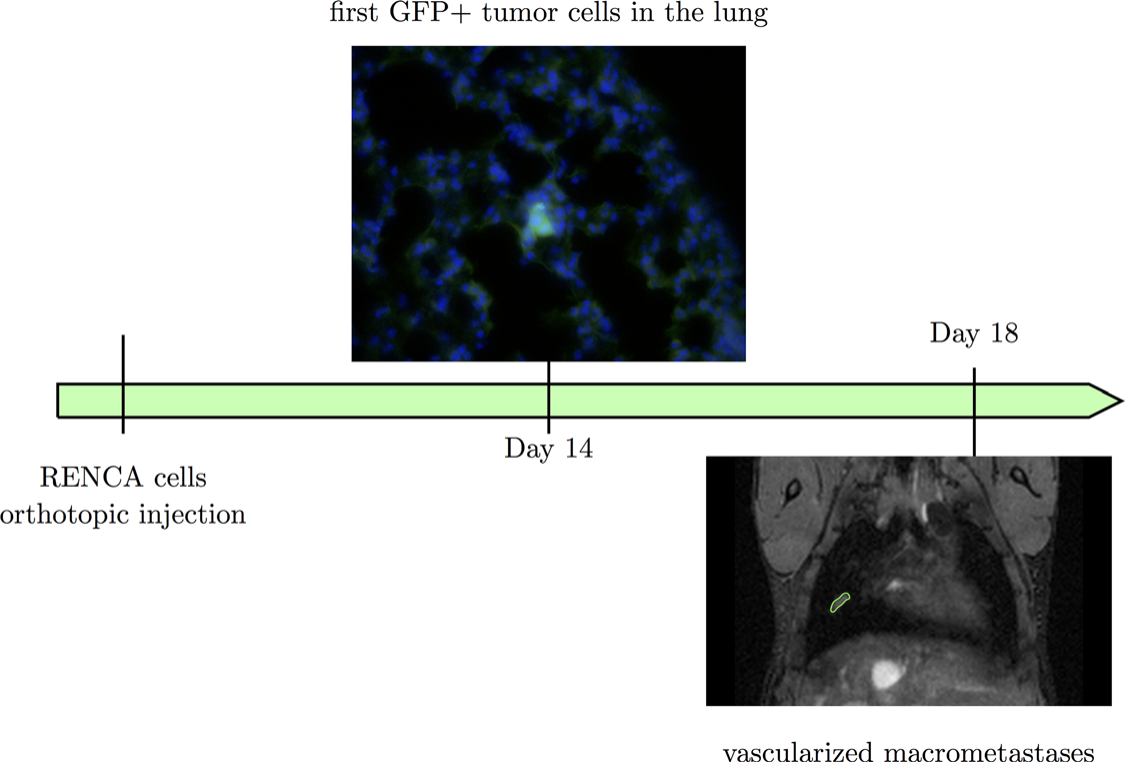
The animal model. At day 14 after GFP+ RENCA cells injection, the first tumour cells were observed in the lungs (in green). At days 18–19, the first macro-metastases were observed by MRI.

RENCA cells were injected orthotopically in the sub-capsular space of the kidney of Balb/c mice. The first metastatic cells were observed in the lungs at day 14 and the first macro-metastases at days 18–19 (Fig 1). No metastasis was observed in other organs.

Assuming in a theoretical model that each metastasis originates from one surviving cell would imply that some metastases grow from the volume of one cell (≃10^−6^ mm^3^, according to the well-established conversion rule 1 mm^3^ ≃ 10^6^ cells [29]), to a volume of few mm^3^ (between 0.022 and 12 mm^3^) in five days at most. This would give tumour doubling times comprised between 5 and 8 hours, which represent less than one third of the doubling time observed *in vitro* (24.5 hours [30]). Even if considering that the metastases arose from few cells (2–50) instead of one [31,32], this would imply doubling times between 5.5 and 13.5 hours. These doubling times would also have to remain constant during 5 days. Such a fast growth is highly improbable since no mammalian cell has a cell cycle length smaller than 10 hours [33]. Moreover, the doubling time has been reported to be non-constant and to increase during *in vivo* growth [15]. Hence, growth at initiation would have to be even faster, in order to fit the data. Therefore, the theory consisting in describing each metastasis with a tumour expansion only based on cell proliferation seems unlikely.

#### Primary kidney tumour and the dynamics of lung metastasis

**Fig 2:**
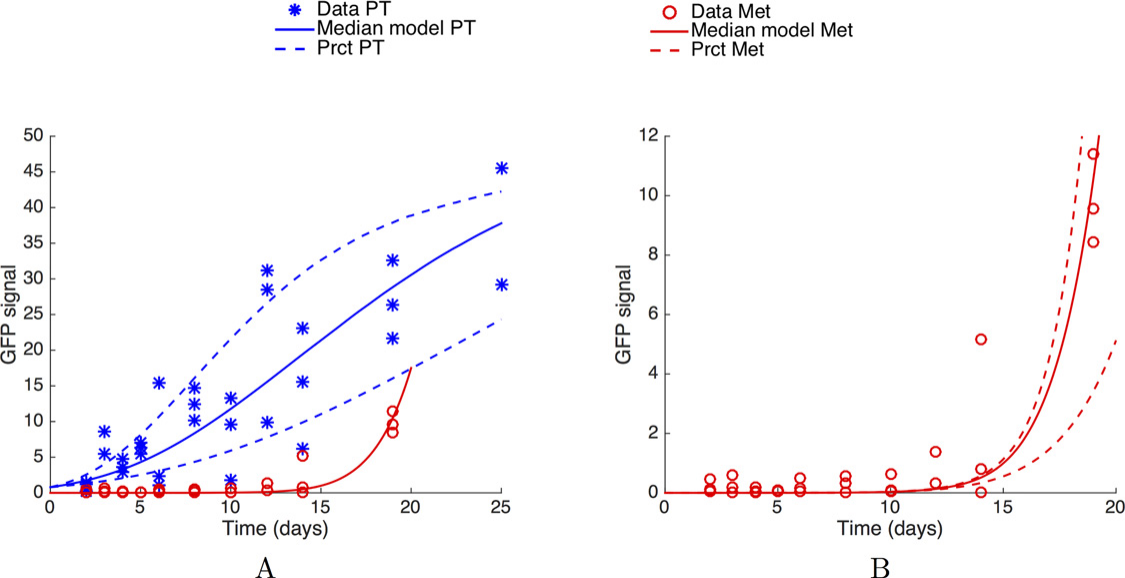
The standard theory: Primary tumour and metastatic burden dynamics fitting. (A) Fits of the primary tumour and metastatic burden dynamics, under a mathematical model assuming independent growth of each secondary tumour and using mixed-effects modelling for statistical representation of the population distribution of the parameters and measurement error. (B) Fit on the metastatic burden. In panels (A) and (B), each data point corresponds to one distinct mouse (*n* = 31 animals in total). Simulations were obtained using Eq 1 for the primary tumour growth and Eq 3 for the metastatic burden, endowed with a lognormal distribution of the parameters with the following values (median ± standard deviation): λ = 0.679 *α* = 0.417 ± 0.171 day^-1^, *β* = 0.106 ± 0.0478 day^-1^ and *μ* = 9.72 × 10^−6^ ± 0.428 × 10^−6^ cell∙day^-1^. PT = Primary Tumour. Met = Metastatic burden. Prct = 10% and 90% percentiles

The standard theory of metastatic development assumes that secondary tumours are seeded from the primary tumour and that, once established at the distant sites (the lungs in our case), they grow independently from each other and from the rest of the organism [2,3,10,34–36], as distinct foci initiated by single or few cells. We tested this theory against the following data: a) dynamics of the increase of GFP+ tumour cells in the kidney, b) longitudinal quantification of GFP+ tumour cells in the lungs and c) MRI images of lung metastases. To this aim, we formalized the standard theory into a mathematical model. The model was then fitted to the data a) and b) and predictions were compared to the data c). For data a) and b) (Fig 2), quantification of GFP expression by quantitative real-time polymerase chain reaction (see Materials and methods) required sacrifice of the animals. Therefore, each data point corresponds to a distinct animal (*n =* 31 animals in total). Since tumour cells were not detected in the lungs before day 14, measured GFP signals in the lungs in the first days were considered as background noise. Including very early time points for the fit would therefore result in a strong bias because the model would be fitted on GFP values that do not reflect the presence of tumour cells. Thus, we considered the data points only starting from day 10 for the fit and ignored the previous data points. At day 25, the GFP signal in the lungs of the two mice sacrificed at this time point was within the noise level. Considering as highly unlikely the event of no metastases at such an advanced time, particularly when observing that imaged mice at day 24 either exhibited lots of metastases in the lungs or were dead due to metastatic disease, we concluded to a technical issue and removed these two data points from the analysis.

The standard theory as described above has been mathematically formalized by Iwata et al. [26]. This model was reported to provide a valid description of the dynamics of total metastatic burden in two animal models, including ours (human breast carcinoma xenograft [27,28] and syngeneic renal cell carcinoma [28]).

The model was adapted here as follows. We assumed that the GFP signal was proportional to the number of cancer cells, itself proportional to the tumour volume observed by MRI. The primary tumour volume at time *t* was denoted *V*_*p*_(*t*) and its growth rate *g*(*V*_*p*_(*t*)). The primary tumour disseminates metastatic cells into the lungs according to a volume-dependent dissemination law *d*(*V*_*p*_(*t*)). The metastatic colonies then grow into the lungs according to the same growth law as the primary tumour. The model describes the size distribution of the metastatic lesions at the distant site by means of a size-dependent density *ρ*(*t*,*v*) of metastatic colonies of size *v* at time *t*, i.e *ρ*(*t*,*v*)*dv* is the number of metastatic colonies with a size comprised between *v* and *v* + *dv*. Secondary emission of metastases (i.e., metastases from metastases) was neglected here.

Tumour growth was modelled by means of the Gomp-Exp model [37], which is characterized by two phases: first an exponential phase (with a growth rate given by the *in vitro* proliferation rate), then a Gompertz phase (i.e., exponentially decreasing growth rate). For the primary tumour growth, the model writes

$$\{\begin{array}{c} \frac{dV_{p}}{dt}\left(t\right)=g\left(V_{p}\left(t\right)\right)\\ V_{p}\left(0\right)=V_{inj}\\ g\left(V_{p}\right)=\min\left(\lambda V_{p},\left(\alpha -\beta ln\left(V_{p}\right)\right)V_{p}\right) \end{array}$$ (1)

where *λ* is the *in vitro* proliferation rate of RENCA cells (retrieved from [30]), *α* corresponds to the specific growth rate at the size of one relative unit of GFP signal, *β* is the rate of exponential decrease of the specific growth rate and *V*_*inj*_ is the amount of injected cells. The Gompertz model has been proven able to describe in vivo tumour growth in numerous animal experimental systems [15,38,39], as well as human data [40]. Adjunction of the initial exponential phase was considered here because the Gompertz model exhibits an infinite relative growth rate for arbitrary small cell numbers, a feature that was not considered relevant, especially for the metastases that start from one cell. At this point we needed to retrieve the GFP signal associated to one cell. To achieve this, we performed a preliminary fit of the primary tumour GFP signals with the parameter *V*_*inj*_ subject to optimization, using population mixed-effects statistical modelling (see S1 Fig). We then assumed proportionality between the number of cells injected (100 000) and the estimated signal at day 0. The GFP signal associated to one cell was then derived and is denoted by *V*_0_. We found *V*_0_ = 7.96 × 10^−6^ relative units of GFP signal.

Growth of secondary tumours was assumed to follow the same law (Gomp-Exp) and parameter values. More complex modelling including different growth parameters for the primary tumour and metastasis were tested but did not substantially improve the fits, while increasing the uncertainty in parameter estimation due to increased number of degrees of freedom.

At present time, there is no detailed study on the shape that the function *d* should have. However, it is often assumed to follow the law [26–28]:

$$d(V_{p})=\mu V_{p}^{\gamma }$$

where parameter *μ* is related to an intrinsic (genetic) probability of the metastatic potential of the tumour cells, combined to the probability of successfully passing all the steps required for establishment of a metastasis (detachment from the primary tumour, intravasation into the blood circulation, survival in transit, arrest and extravasation at the distant site, establishment of a new colony [2,12]). Precise identification of the value of parameter *γ* was not possible on our data set, and in the following analysis, we arbitrarily fixed *γ* = 1, corresponding to the simplest assumption (emission proportional to the tumour volume).

Overall, the model writes as a transport equation on *ρ*, endowed with suitable boundary and initial conditions [26]:

$$\{\begin{array}{c} \begin{array}{cc} \partial_{t}\rho \left(t,v\right)+\partial_{v}\left(\rho \left(t,v\right)g\left(v\right)\right)=0 & t\in \end{array}]0,+\infty \left[,\;v\in \right]V_{0},+\infty [\\ g\left(V_{0}\right)\rho \left(t,V_{0}\right)=d\left(V_{p}\left(t\right)\right)\\ \rho \left(0,v\right)=0 \end{array}$$ (2)

From the solution of this problem, one of the main quantity of interest for our purpose was the total metastatic burden, defined by

$$M\left(t\right)=\int_{V_{0}}^{+\infty }v\rho \left(t,v\right)dv$$ (3)

The model output was fitted to the GFP expression data from the lungs (Fig 2).

Due to the large inter-animal variability, we used a nonlinear mixed-effects statistical framework for fitting the model to the data and estimation of the parameters [41] (see also [27,28] for applications to bioluminescence data of metastatic burden), which is particularly well suited for sparse longitudinal data in an animal population. Briefly, this framework considers estimation of a (parametric) distribution of the parameters within the population. This allows pooling all the data points together, thus leading to an increase of the robustness of the estimation and of the descriptive power for inter-animal variability. For the maximization of likelihood associated to nonlinear mixed-effects modelling, we used a version of a stochastic expectation maximization algorithm implemented in the Matlab function *nlmefitsa* [42]. To simulate the model, a well-adapted method using an integral formulation for *M*(*t*) and the fast Fourier transform algorithm was employed [43]. This ensured reduction of the computational cost of simulations, which was necessary due to the very high number of runs required by *nlmefitsa*.

Data from the primary tumour and the metastatic burden were fitted together, and the model demonstrated satisfactorily descriptive power for the total metastatic burden (Fig 2), in accordance with other studies [27,28]. Parameters resulting from the fit procedure are reported in Table 1.

The calibrated model was further used to predict the distribution of macro-metastases visible in the MRI images, and to confront this prediction to the observations. Among the MRI data, images of only one mouse (over 6) were eligible for reliable assessment of the complete size distribution of macro-metastases, which was performed by manual segmentation of metastatic lesions in each of the 142 coronal slices of the MRI (resolution 156 μm x 155 μm 155 μm), for each time point. In the other mice, the images had no sufficiently defined contours to properly establish a complete size distribution of the metastases (see S2 Fig). However, segmentation of the largest metastasis for each mouse at day 19 could be performed.

**Fig 3:**
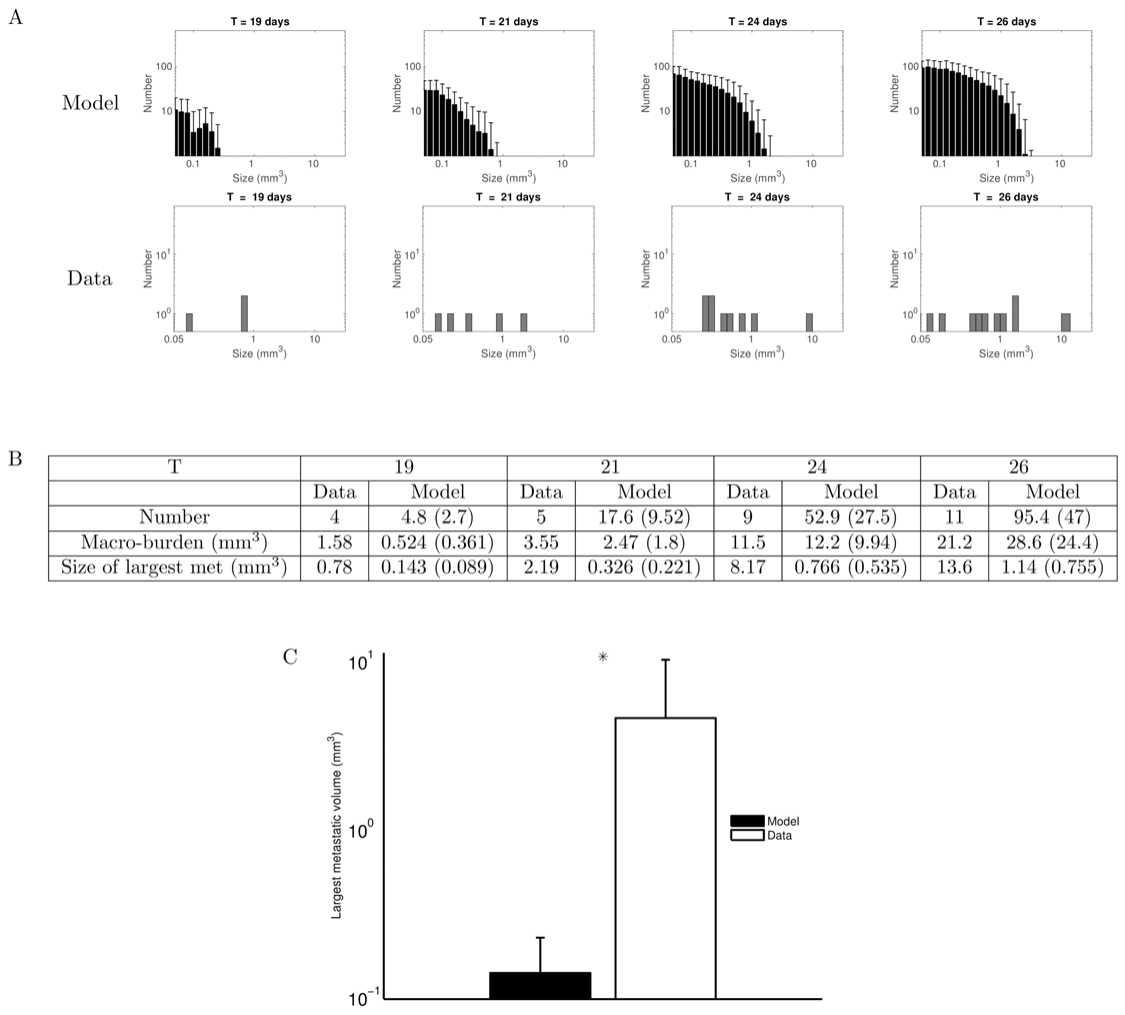
Time course of the macro-metastases size distribution: standard model versus observations. (A) Top row: Simulation of the mathematical formalism of the standard theory (i.e. dissemination and independent growth of the resulting tumour foci), using the parameter values inferred from the data of the total metastatic burden (total GFP signal in the lungs). Only tumours larger than the visible threshold at MRI (0.05 mm^3^) are plotted. Simulations were obtained using Eqs 1 and 2 for the time evolution of the density of secondary tumours, endowed with a lognormal distribution of the parameters for inter-animal variability, with the following values (retrieved from the population mixed-effects fit, median ± standard deviation): λ = 0.679 *α* = 0.417 ± 0.171 day^-1^, *β* = 0.106 ± 0.0478 day^-1^ and *μ* = 9.72 × 10^−6^ ± 0.428 × 10^−6^ cell∙day^-1^. Shown are the results of 1000 simulations, mean + standard deviation. Bottom row: Observations of macro-metastases numbers and sizes in one mouse on MRI data. (B) Comparison of several metrics derived from the metastatic size distributions. For the model, numbers are represented as mean value and standard deviation in parenthesis. The data corresponds to the mouse presented in the upper histogram. (C) Comparison of the largest metastatic size at day 19 between model (*n =* 1000 simulated animals) and observations (*n =* 6 animals), log scale. The observed largest metastases are significantly larger than simulated ones (*p* < 10^-5^ by the z-test).

In the mouse where number and size of the lesions could properly be assessed, the smallest detectable metastasis had a volume of 0.05 mm^3^, which was taken as the minimal visibility threshold. We defined a macro-metastasis as a metastasis having a size larger than this value. Results of the model simulation for the metastatic size distribution are reported in Fig 3A, together with the experimental data. Inter-animal variability was simulated using population distribution of the parameters (lognormal distribution and coefficients of variation reported in Table 1), retrieved from the population mixed-effects fit. The maximal volumes predicted by the model/standard theory were considerably smaller than those observed by MRI. For example, at *T = 19* days, while the total metastatic burden was similar in the data and in the model (Fig 2), the macro-metastatic burden was three-fold larger in the data than in the model’s average prediction (Fig 3B), and the largest metastasis five-fold larger. At *T = 26* days, although macro-metastatic burdens were similar in the data and in the model, the standard theory predicted that the largest tumour would have a volume of only 1.14 mm^3^ in average (standard deviation = 0.755 mm^3^), while the largest observed metastasis had a volume more than 10 fold larger (13.6 mm^3^). This was compensated by a considerably larger number of metastatic lesions in the model (95.4 ± 47 versus 11 in the data). For each of these quantities, the *p*-value (probability to obtain the data value–or larger–under the null hypothesis that the data would have been generated by the model, evaluated numerically) was statistically significant (*p <* 10^−5^).

**Table 1:** Parameters values resulting from the population fit of the primary tumour and metastatic dynamics.

Par.	Unit	Median value (CV %)	95% CI
*α*	day^-1^	0.417 (41)	(0.329–0.557)
*β*	day^-1^	0.106 (45.1)	(0.0372–0.145)
*μ*	cell^-1^day^-1^	9.72 × 10^−6^ (21.7)	(1.81–52.2) × 10^−6^

CI = confidence interval.

CV = Coefficient of Variation, in per cent = $\frac{std}{est}\times$100, with *est* and *std* respectively the median value and standard deviation of the estimated lognormal population distribution of the parameters resulting from the nonlinear mixed-effects statistical estimation procedure.

These conclusions are limited by the fact that the entire time course of metastatic size distribution of only one mouse was available for reliable comparison with the model. However, in all the 6 mice, the size of the largest metastasis at day 19 could be measured and ranged 0.45-12 mm^3^, which was significantly larger than the model predictions (Fig 3C, *p <* 10^−5^ by the z-test). To give an idea, the largest metastases predicted by the model ranged 9.5x10^-4^ – 0.3 mm^3^. This strongly suggested that the standard theory was not able to describe the volumes of individual foci. Moreover, even without statistical comparison of the model’s predictions to the empirical data, the numbers predicted by the model (in particular the number of macro-metastatic lesions at day 26) seem highly unrealistic.

To assess the robustness of our results regarding several assumptions of the model, we investigated varying several parameters. First, metastases might initiate from a size larger than from one cell [31,32]. We therefore performed the entire analysis for different values of *V*_0_ (see discussion and S3 Fig), and found similar inconsistencies with the data in terms of largest metastases. Data-consistent and biologically plausible results in terms of number of metastases would require initial sizes larger than 100 cells, which is biologically unrealistic in view of the size of capillaries and experimental works that demonstrated that tumour cell clumps comprise less than 10 cells [31]. Similar results demonstrating inconsistency of the standard theory were also obtained when re-performing the analysis for variable values of the parameter *γ* (S4 Fig).

These results strongly indicate that the standard theory of metastatic progression as described by the model employed here (i.e., dissemination and independent growth), when calibrated to data of total metastatic burden, was in contradiction with the experimental observations with regard to the number of metastatic foci and their size distributions.

**Fig 4:**
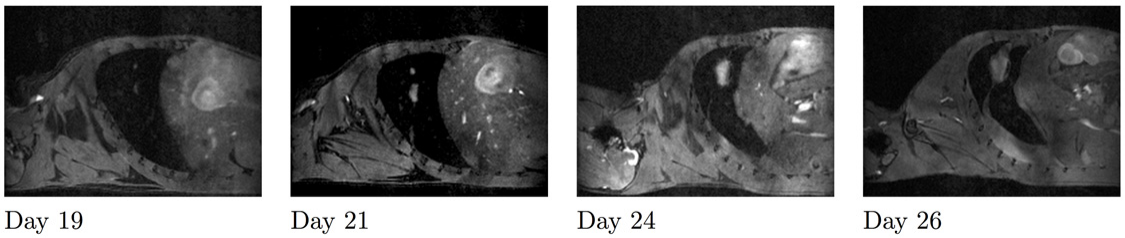
Metastases merging. From left to right: Sagittal slices of the lungs from day 19 until day 26 for the same mouse. Two tumours are growing close to each other and merge between days 21 and 24.

It is beyond the scope of the present work to elaborate (and validate against the data) a unified model able to recapitulate the behaviour of metastatic tumours during the colonization process. However, as a first step toward this objective, we put forward two assumptions to correct the inconsistency of the standard theory: (1) non-trivial interactions between metastases and (2) interactions between the metastatic foci and the circulating tumour cells (cells attraction). We indeed observed merging of two metastases in our data (between days 21 and 24, see Fig 4) and therefore decided to investigate this further. More specifically, we wanted to address the following questions: do spatial interactions have an impact on the dynamics of the total metastatic burden? To what extent could this correct the theoretical predictions of the unlikely fast growth rates? Answers to these questions have implications on future theoretical models of metastatic development. The possibility of merging for two neighbouring metastases introduces a spatial aspect of metastatic colonization and, therefore, requires a spatial modelling approach. We derived such a model which had to full-fill the following requirements: 1) it should be based on biological knowledge of macroscopic tumour growth (retrieved from the literature), 2) it should remain as parsimonious as possible (minimal number of parameters) and 3) it should be able to fit our spatial growth data.

### Spatial interactions between metastatic tumours

#### Advection-type modelling of growth and movement

The two-dimensional model we used for the spatial growth describes a saturated flow in a porous medium comprising two entities, the tumour tissue and the healthy tissue, with *P* denoting the tumour cell density and *S* the healthy cell density. The third variable is a pressure field *Π*. The model describes, on a domain *Ω*, the passive motion of the tissues due to the increase in volume caused by proliferation. Writing a mass-balance equation, this corresponds to the following:

$$\frac{\partial P}{\partial t}\left(t,x\right)+\nabla .\left(v\left(t,x\right)P\left(t,x\right)\right)=\gamma \left(t,x,P,S,\Pi \right)P\left(t,x\right)$$ (4)

$$\frac{\partial S}{\partial t}\left(t,x\right)+\nabla .\left(v\left(t,x\right)S\left(t,x\right)\right)=0$$ (5)

where γ is the growth rate of the proliferating cells (see below for its expression). We also assumed that the flow is saturated, with *P* + *S* = 1 at each point of the domain. Summing (4) and (5) leads to the following condition on the velocity of the flow:

∇.v=γP (6)

This condition means that the tumour volume changes are due to proliferation. We considered the organ (the lungs) as a porous medium with a porosity *k*(*t*, *x*) and that the velocity *v* is due to pressure gradients within the tumour. Therefore, we modelled the velocity flow by a Darcy law, as Preziosi and Ambrosi in [44]:

v=−k∇Π (7)

The velocity *v* is the passive motion velocity, due to the pressure exerted by the proliferative tissue on the surrounding tissues. These tissues are in this way "pushed" and move away from the high-pressure areas to the lower-pressure ones.

The size of the computational domain *Ω* was fixed to the order of magnitude of mouse lungs (≃ 1 cm^3^). Assuming that no mechanical interactions occur with the organ boundaries (for instance, the possible deformations of the organ due to the growth are neglected here), we supposed the domain *Ω* large enough to consider the pressure on the boundaries as equal to the homeostatic pressure of the body. We model this by a Dirichlet boundary condition on the pressure:

$$\Pi =\Pi_{eq}\;\mathrm{on}\;\partial \Omega$$

Collecting (6) and (7), and considering the porosity *k* constant, we obtain *Π* to satisfy a Poisson equation with Dirichlet boundary conditions:

$$\{\begin{array}{c} -k\Delta \Pi =\gamma P\\ \Pi_{|\partial \Omega }=\Pi_{eq} \end{array}$$

In this model, taking γ constant leads to exponential growth of the tumour burden. However, *in vivo* growth can depend on environmental conditions, leading to increased doubling time when the conditions are not optimal. Similar models using more cellular species were used by Ribba, Colin and Schnell in [45] to predict efficacy of radiotherapy, and by Colin, Saut and colleagues in [46] to describe avascular tumour growth. In the latter work, lack of nutrients and hypoxia were considered as essential limiting factors for growth and hence included in the model, thus introducing a supplementary variable (nutrients concentration or the vasculature density). However, in our study, because we wanted to keep the model as parsimonious as possible, we focused on a more phenomenological way to describe the fact that the tumour doubling time increases with the tumour size. The natural environmental variable being the pressure, a simple way to formalize this was to model the proliferation rate as a decreasing function of the pressure. This led to a simple model that captured the essential features of tumour expansion and was able to describe *in vivo* tumour growth.

#### Modelling the pressure-mediated inhibition of tumour growth

Following the work of Montel et al. [47] and Stylianopoulos et al. [48,49], we considered that the growth rate of the tumour tissue decreases with the pressure exerted on the tissue. Therefore, we modelled the growth rate with a decreasing exponential law [47]:

$$\gamma (\Pi )=\gamma_{0}\exp\left(\frac{-\Pi }{\Pi_{0}}\right)$$

where *Π* represents the pressure field, *γ*_0_ the maximal proliferation rate, and *Π*_0_ a characteristic pressure. Under the assumption of a constant porosity, the value of *k* has no impact. Indeed, as long as the product *kΠ*_0_ remains constant, the solution remains unchanged. That is why we fixed k=1. Moreover, the boundary condition was taken homogeneous: *Π*_*eq*_ = 0, which means that the homeostatic pressure of the body is the optimal pressure of proliferation.

Under such a model, high pressure provokes decreased proliferation, but not apoptosis. Montel et al. suggested that mechanical stresses have a poor effect on apoptosis, but also indicated that this could depend on the cell line and the experimental protocol [47].

#### Model calibration

**Fig 5:**
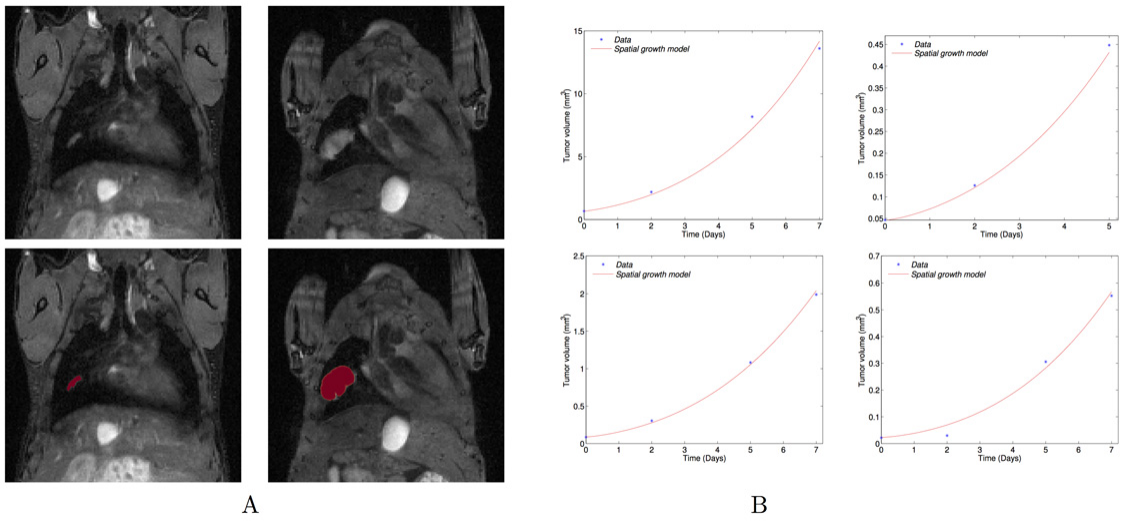
Spatial model fitting. (A) Top: Coronal MRI data of the lungs at days 19 and 26. Bottom: the simulated growth by the model using the fitted parameters and starting from the real shape of the observed metastasis at day 19 on the coronal MRI slice. Simulations were obtained using Eqs 4–7 with the following parameter values: *γ*_0_ = 0.78 day^-1^; *Π*_0_ = 0.0026 Pa; Time of simulation: T = 7 days (B) Volumes compared to simulations by the fitted model for the growth of four individual metastasis. The fits were performed on the volume only, considering the metastases as spherical.

To perform the study, we first wanted to fix the parameters of the model to realistic values. We calibrated the model according to the growth of four metastases observed by MRI. These four tumours were selected because they were detectable during sufficient time points (four for three of them and three for one of them) and were manually segmented. Fig 5 shows the MR images, the numerical simulation starting from the initial shape with the parameters fitted to the volume for one of the four metastases, and the dynamics of the simulated volume changes of the four metastases. A movie of the simulation on the MR image is shown in the supplementary S1 File and spatial distributions of pressure (Π) and proliferation rate (γ) are presented in S5 Fig. The model was able to describe the increase of the tumour volume for the four metastatic lesions with excellent goodness-of-fit. Table 2 presents the values of the two parameters for the four fitted growth curves. The fits were performed on the volume only, considering the metastases as spherical, which is a reasonable assumption because only slight differences on the mass are observed between the spherical and non-spherical cases (S6 Fig).

**Table 2:** Values of the parameters resulting from the fit of each metastatic dynamics.

	*γ*_0_ (day^-1^)	*Π*_0_ (Pa)
Meta 1	0.78	0.0026
Meta 2	1.01	0.00079
Meta 3	0.67	0.00067
Meta 4	0.8	0.00052

The value of *γ*_0_ corresponds to the maximal proliferation rate in optimal conditions of pressure.

These fitted parameters provided a range to perform the tumour-tumour contact interactions study:

$$\left(\gamma_{0},\Pi_{0})\in (0.67,1.01)\times (5.2\cdot 10^{-4},2.6\cdot 10^{-3}\right)$$

#### Quantitative impact of pressure-mediated growth interactions

We next aimed at studying the quantitative impact of the pressure that two neighbouring metastases exert on each other when they grow, and whether the merging hypothesis could explain the fast metastatic growth rates.

**Fig 6:**
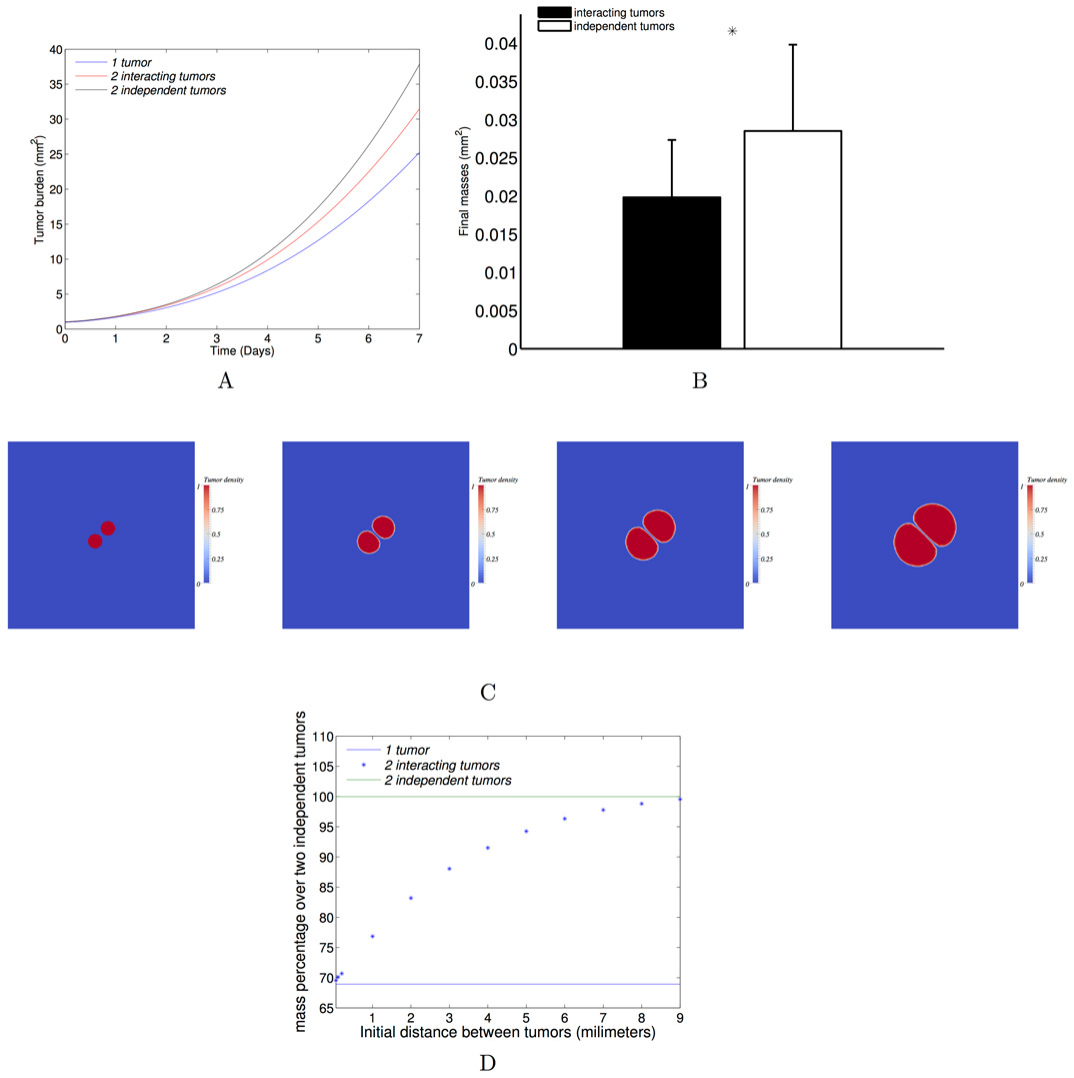
Tumour-tumour spatial interactions. (A) Three different configurations with a same initial burden: only one tumour, two close tumours, two far tumours. The dynamics in the three configurations are compared with the parameter set inferred from the fit on one metastatic growth (0.78, 0.0026) day^-1^×Pa. (B) The final burdens are compared in two configurations: two close tumours and two independent tumours. The mean burdens over a set of 64 parameters (resulting from an 8 × 8 uniform discretization of the relevant parameter space given by the individual tumour fits, (0.67,1.01) × (5.2 ∙ 10^−4^,2.6 ∙ 10^−3^)) are plotted with the standard deviations (difference of 31% ± 1.5% between the two distributions). (C) From left to right: time course of two interacting tumours growing and pushing each other. The parameters were fixed from one of the fitted MRI metastases: *γ*_0_ = 0.78 day^-1^; *Π*_0_ = 0.0026 Pa; simulation time: T = 7 days; initial distance between the two metastases: D = 0.2mm; initial surface for each metastasis: S = 0.46 mm^2^. (D) The curve represents the evolution of the final burden with respect to the initial distance between the two interacting tumours. The initial total burden and the parameters were taken to be the same as one of the four fitted metastases (same as C).

A first simulation of two interacting tumours was performed by choosing a bi-focal initial condition to Eqs 4 and 5, and using the parameters of Meta1 (*γ*_0_ = 0.78, *Π*_0_ = 0.0026, see a movie in the supplementary S2 File). When comparing the total growth (sum of the surfaces of the two tumours) to the growth of only one tumour seeded with the same initial surface as the two tumours together, we observed a slower growth in the single tumour (Fig 6A). Indeed, in a larger tumour, there are more cells that proliferate, resulting in higher mechanical constraints than in a smaller one. The pressure is therefore higher in the larger tumour, resulting in faster saturation of tumour growth over time.

To quantify the impact of the mechanical interactions, we then compared the two following situations: (1) two metastases that grow independently (the final burden consists in summing-up the two burden) and (2) two metastases that are close to each other (the exerted pressure of one metastasis impacts the growth of the other).

The two configurations were studied and compared with 64 sets of parameters chosen in the parameter space established by the calibration. The results highlight the possibility for two metastases to mutually impair their growth by mechanical interactions. Indeed, by proliferating, the neighbouring tumours exert pressure on each other, which leads to a decrease in proliferation in comparison to distant growing tumour foci. More precisely, under the assumption of increasing doubling time with respect to the pressure, the calibrated model revealed substantial differences in tumour burden, as shown in Fig 6B. Among all the parameter sets, when two tumour foci interact, at the final time (*T = 7* days, which corresponds to the time scale of the four metastatic growths) the loss of mass was 31% ± 1.5% (mean ± standard deviation) in comparison to distant growing tumour lesions. As an example, Fig 6C presents a simulation of two interacting tumours at four time points.

We observed in our data neighbouring metastases growing close to each other until merging (Fig 4). To simulate merging of two metastases, we did not introduce any merging effect in the model. It occurred naturally in the model when the tumour density field consists of two tumour foci growing in close vicinity. Mechanical interactions occurred at the time of merging, resulting in a slow-down of tumour growth. In terms of global dynamics, two different merging times generated two different dynamics. This merging time is equivalent to the initial distance between the two metastatic foci. We therefore studied the impact of the initial distance. Under our modelling assumptions, the interactions between two metastases decrease with the initial distance between them (Fig 6D). This means that the later the metastases merge, the larger the final burden is. When the initial distance between the tumours goes to 0, the burden corresponds to the burden of only one tumour. When the distance tends to infinite, the burden is equivalent to the burden of two independent tumours.

We studied the effect of the fractionation of a same burden for independent metastases and for interacting metastases with a distance of 0.2 mm between metastases. S7 Fig depicts the evolution of the burden as a function of the number of tumours in the case of independently growing tumours or tumours that mechanically interact. As shown, the difference between both situations increases with the number of metastases. For instance, for 18 metastases growing close to each other, the loss of mass from the independent case to the interactions case is 76.3% (to be compared to the 31% for two tumours).

#### The merging hypothesis

**Fig 7:**
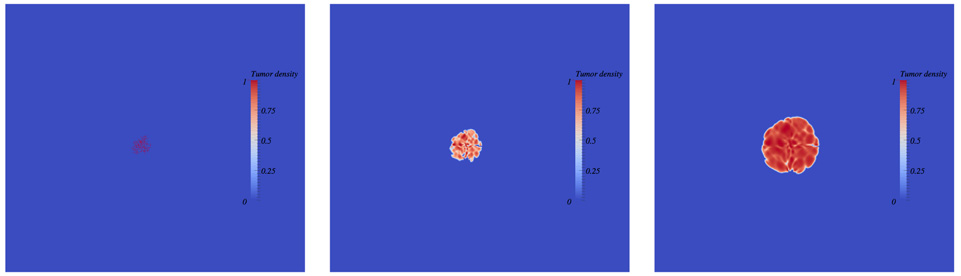
Simulation of multiple metastatic foci merging (with spatial interactions). From left to right: time course of merging metastatic germs. Each germ starts from one cell. The germs are randomly located at a distance of 0.03 mm from each other. Simulations were obtained using Eqs 4–7 and the following parameter values: *γ*_0_ = 0.78 day^-1^; *Π*_0_ = 0.0026 Pa; time of simulation: T = 7 days; number of germs = 200 in 2D. The corresponding number of cells in 3D is computed under a spherical symmetry assumption and is 2127. Movie of the simulation is available as S3 File.

We investigated whether the merging of metastatic foci could have generated the formation of macro-metastases in the required timeframe, with biologically realistic growth rates. We investigated the two situations: without spatial interactions (i.e., assuming the volume resulting from the merging as equal to the sum of the metastatic foci volumes), and with spatial interactions. To do so, we performed four simulations with the four fitted parameter sets, starting from one cell, to estimate the number of merging metastatic foci required to obtain the respective observed volumes (of 0.022, 0.046, 0.085 and 0.67 mm^3^) seven days after initiation (day nineteen). Indeed, we chose day twelve and not day fourteen (which was the time at which the first metastatic cells were observed by direct examination of lung tissues) as the starting day because the GFP signal started to rise at day twelve (Fig 2). The required numbers are presented in Table 3, with and without spatial interactions between the tumours. For the spatial interactions case, simulations were performed as follows. Each focus was assumed to start from one cell and the foci were randomly distributed within the computational domain. To avoid metastatic foci too close from the domain boundaries and to allow the foci to merge together, the initial distance between the foci was constrained to 0.03 mm. Fig 7 depicts the simulations results (see supplementary S3 File for a movie). The number of required metastases reported in Table 3 has been estimated by dichotomy (the final burden increases with the number). Because the initial distance between the foci was small, the mechanical interactions were maximal here. The estimated number is therefore probably overestimated. Consequently, the two estimated numbers (with and without spatial interactions) give an approximate range for the exact required number.

**Table 3:** Number of required merging foci.

	Required number of metastases	
	Without spatial interactions	With spatial interactions
Meta 1	1337	2127
Meta 2	20	65
Meta 3	301	375
Meta 4	40	70

There it is the number of required merging foci to obtain the metastatic sizes measured on the MR images for each followed metastasis. Two cases are considered: with and without spatial interactions.

As can be seen in Table 3, since spatial interactions reduce the growth velocity, the number of metastases was higher when interactions were taken into account. Because of potential variability (error measurements during the segmentation, differences between the MRI signal and the real lesion, especially for the small metastases, modelling assumptions), the estimated numbers of required metastatic foci may give only a rough estimate. For two of the metastases (Meta 2 and Meta 4), the estimated numbers appear to be reasonable. On the other hand, for the two other ones, the required number ranged respectively between 301 and 375 and between 1300 and 2100, which are probably too large to be biologically realistic.

Besides spatial interactions, another possible phenomenon involved could be the attraction and aggregation of circulating tumour cells. This hypothesis is discussed below.

## Discussion

Using a combined approach between experimental data and mathematical models, we demonstrated that the standard theory of metastasis formation and growth, where metastases grow independently from the rest of the system, was biologically unlikely. To explain our findings, we proposed several hypotheses, including the possibility of metastatic foci merging by passive motion. To investigate whether this hypothesis would have quantitatively non-negligible impact on the kinetics of the total metastatic burden (thus requiring more intricate modelling for the model describing the size distribution at the scale of the organ), we introduced a parsimonious spatial model of tumour growth. After calibrating the model to the growth of single metastases, we found (in simulations) that spatial interactions resulted in a significant reduction of tumour growth. Our results indicate that spatial interactions should be considered in future efforts for the development of a general quantitative theory of metastatic colonization.

Based on the rationale that lung capillaries have a diameter of the order of one tumour cell (20 μm) and that metastatic cells have lost expressions of cell-cell adhesion proteins such as cadherins [2], we assumed in our simulations, that metastases originated from one cell. This might be arguable and metastasis could start from tumour cell clumps [31,32]. To resolve this further and assess the robustness of our results, we performed the entire data analysis (fit of the total metastatic burden and resulting prediction of the metastatic size distribution) for values of the initial number of cells of 1, 10, 100 and 500 (S3 Fig). Initial numbers of 10, 100 and 500 cells could be in agreement with the data at day 19. However, with *V*_0_ = 10 cells, the predicted number of macro-metastases at day 26 was 3-fold higher than in the data. For *V*_0_ = 100 cells and *V*_0_ = 500 cells, the predicted macro-burden was 2-fold smaller than the observed one. Moreover the largest metastasis at day 26 was still predicted much smaller in the model than in the data (3.11 mm^3^ for *V*_0_ = 10 cells, 3.58 mm^3^ for *V*_0_ = 100 cells, 3.8 mm^3^ for *V*_0_ = 500 cells, against 13.6 mm^3^ in the data). Furthermore, in animal experiments the vast majority of detaching tumour cell clumps has been shown to comprise less than 10 cells [31] with a range of 2–50 cancer cells [32], which makes the theories *V*_0_ = 100 cells and *V*_0_ = 500 cells unlikely. This suggests that, if the metastases started from a substantial amount of cells, the grouping of these cells probably occurred at the distant site, after extravasation from the blood circulation. Similarly, we did not consider any cell loss at the moment of initial sub-capsular injection. We could make theoretical assumptions of cell loss (of 10%, 20%, etc…), which would simply consist in multiplying *V*_0_ by the relevant factor. For instance, considering a 90% loss (i.e. that only 10% of the cells remain viable) would be equivalent to multiplying *V*_0_ by 10. As demonstrated in S3 Fig, it is necessary to assume an initial size of at least 100 *V*_0_ to recover plausible values for the number of metastases at time *T* = 26 days. Combining the two (cell loss of 10% and initial metastatic size of 10 cells) thus gives a hypothesis that we are not able to infirm given the data we dispose.

The spatial model for tumour growth that we introduced is based on a pressure-induced decrease of the growth rate. Contact inhibition between cells is a mechanism for maintaining tissue homeostasis [4]. The ability of cancer cells to ignore these inhibition signals is a hallmark of cancer. In a recent study, Stylianopoulos et al showed that the uncontrolled proliferation of tumour cells results in mechanical stresses in the surrounding micro-environment of transplanted and human tumours [48]. Furthermore, they also showed that such an exerted pressure impairs *in vivo* proliferation via two mechanisms: reduced cancer cell proliferation in direct response to increased pressure, as well as a pressure-induced collapse of blood vessels within the tumour, leading to nutrient deficiency for tumour cells [49]. Based on these considerations, it seems relevant to consider that tumour expansion depends on the pressure. In our spatial growth model, the tissues motion is mediated by pressure gradients. It means that cells within a tumour tissue proliferate and that the exerted pressure pushes the neighbouring tissues. This pressure is not solely due to mechanical constraints (solid stresses, interstitial fluid pressure,…) exerted by the neighbouring cells on each other, but represents a more phenomenological pressure, that reflects the basic assumption of our modelling strategy for the tumour tissue being constituted by a fluid mixture in a porous medium. The effect of the pressure on proliferation has also been studied using numerical simulations elsewhere. In [47], Montel et al discussed the fact that cells proliferate faster on the surface than in the bulk of a tumour spheroid. A classical reason is that nutrients do not penetrate deeply in the spheroid. However, Montel et al. suggested a mechanical effect due to the necessity for a cell to deform its environment in order to proliferate. In an *in silico* study on two-dimensional monolayers and three-dimensional spheroids, based on experimentally determined biophysical parameters, Drasdo and Höhme suggested that pressure conditions have a higher impact on doubling time than lack of nutrients [16]. Moreover, in [47], Montel et al. performed experiments where tumour cells were submitted to different pressure constraints and observed a decrease in proliferation when pressure was applied. In their study, simulation results that were compared to experimental ones showed an exponential decreasing of proliferation with pressure, consistently with the modelling adopted here. However, the bulk and surface division rate were not affected equally by stresses. In our model, we used a similar pressure-mediated proliferation law translating direct effects of mechanical stresses on proliferation as well as indirect effects of proliferation on the micro environment (collapsing of blood vessels leading to lack of nutrients).

Our proposed hypotheses should be further experimentally reinforced, by, for example, implanting orthotopically and injecting intravenously two groups of cells into mice, each group being tagged with a different colour, and by quantifying single or mixed-coloured tumour foci. Similar experimental protocols have been already performed in [7,32]. Furthermore, in vivo investigations by observing two (or more) growing tumours in close vicinity that would enter mechanical interactions and then assess with a Ki-67 staining if the proliferation is impaired in the contact area, would further reinforce our contentions.

The inability of the merging theory to explain all of the observed volumes may indicate that besides merging by passive motion due to proliferation, other mechanisms such as chemokine-mediated cells attraction occur [6,50]. Circulating tumour cells may be attracted by some established niches and explain the abnormally fast volume expansions that we observed. Indeed, such chemokine-mediated attractions are presumed to play an important role for the pre-metastatic and metastatic niches establishment, in mediating myeloid and tumour cells attraction [6,50,51]. Moreover, chemo-attractants may play a role in tissue tropism of metastatic cells [52]. Chemotactic gradients can attract metastatic cells that express the chemokine receptor to specific locations. In the future, additional phenomena such as aggregation and recruitment of cells during the metastatic process from the circulation should be integrated in the standard mathematical model. Another phenomenon that could possibly explain the observed volumes would be the presence of circulating tumour cell clusters that would give rise to metastases [32]. Indeed, Aceto et al. recently showed in a breast cancer animal model that metastases do not originate from single cells only but also from tumour cells clusters that have a higher metastatic potential than single cells. However, they did not show evidence of this phenomenon for kidney cancer and in their experiments, clusters were formed by at most 50 cells. As indicated above, this order of magnitude of the initial cell numbers that colonizes the lung is not able to describe the dynamics of metastasis formation in our model and experimental data.

Taken together, our results indicate that spatial interactions are an essential component for the dynamics of metastasis development in the lung and probably also in other organs. However, it is unlikely that they alone control metastasis expansion. Indeed, when trying to assess whether this concept alone explains the fast growth of various metastases from the beginning of organ colonisation (from the first cell at days 12–14 to 0.022-0.67 mm^3^ at day 19), unrealistic numbers were found for two of the tumours. Thus, other mechanisms are probably also involved such as recruitment of additional cells from the blood stream and micro-environmental cues such as nutrient depletion or responses to environmental stress. Our methodology and results illustrate, furthermore, how a combined approach using multimodal biological data on one hand, and multimodal modelling analysis on the other, provides powerful insights into tumour biology and, in particular, into the metastatic process.

## Materials and Methods

### Ethics statement

Ethical approval for all animal studies was obtained from the Institutional Animal Care and Use Committee of the INSERM Institute in accordance with the National Advisory Committee for Laboratory Animal Research Guidelines licensed by the French Authority. Animal facility: Animalerie mutualisée de Bordeaux 1, authorisation number: B33-522, Date: February 8^th^, 2012. Investigator: Andreas Bikfalvi (authorisation number: R-45GRETA-F1-10).

### Cell line and mouse experiments

#### RENCA-GFP cells

The mouse renal adenocarcinoma cell line RENCA was cultured in RPMI media (Gibco) supplemented with 10% foetal calf serum, 1mM sodium pyruvate, 2mM glutamine, 100U/ml de penicillin and 100μ*g*/*ml* streptomycin., at 37*oC*/5% CO2. RENCA-GFP cells were produced via infection of RENCA cells with a GFP lentivirus, a gift of Dr. C. Grosset (u889 Bordeaux).

#### Orthotopic implantation of RENCA-GFP cells

RENCA-GFP were cultured in exponential growth phase, and harvested by trypsinisation (Gibco). After washing in basal RPMI media, the cells were counted and concentration adjusted to 100000 cells per 25μ*l* in basal media. 25μ of cell suspension was then injected underneath the renal capsule of the left kidney of female Balb/c mice aged 6 weeks.

#### Tissue harvest

Mice were sacrificed at the specified intervals and the left kidney (bearing the primary tumour) and lungs were dissected and snap frozen in liquid nitrogen. RNA was extracted using TRIzol Reagent (Life Technologies) as per the manufacturer’s protocol.

#### Reverse transcription and Q-RT-PCR

RNA samples were quantified using a nanodrop ND-1000 spectrophotometer (Nanodrop Technologies. 1μ*g* of total RNA was reverse transcribed to cDNA using High Capacity cDNA Reverse Transcription Kit (Applied Biosystems). Real-Time PCR was carried out using the Step One Plus Real-Time PCR system. Reactions were carried out in a total volume of 20μ*l* containing 2ng of cDNA, Power SYBR Green PCR Matsermix (Applied Biosystems), and 200nM of each of the forward and reverse primers. The reaction conditions were as follows: 10 mins at 95oC followed by 40 cycles of 15 secs at 95oC and 1 min at 60oC.

Data were analysed using Step One Software v2.3. The housekeeping gene PPIA was used as an endogenous control to nomalize for differences in the amount of total RNA in each sample. Expression of GFP is thus presented as an N-fold difference relative to the total RNA per sample.

The sequences of the primer used was as follows: eGFP: Forward primer 5’-CGACCACTACCAGCAGAACA-3’ Reverse primer: 5’-GAACTCCAGCAGGACCATGT-3’

### MRI material and methods

#### MRI material

The experiments were carried out on a horizontal 7T magnet (Bruker Biospec 70/20, Germany), equipped with a 12 cm gradient insert capable of 660 mT/m maximum strength and 110 μ*s* rise time. Lung imaging was performed using a quadrature emission/reception birdcage coil (inner diameter: 2.5 cm, 5 cm length).

Mice were anesthetized with 1.5% isoflurane in air during the imaging session. Mouse respiration was monitored during the entire experiment by using an air balloon placed on the abdomen (SA Instruments).

#### MRI sequence

A 3D water-selective balanced Steady State Free Precession sequence was used [53]. This sequence induces a T2-like contrast, allowing the detection of metastases with hyper-intense signals without injecting contrast agents [54]. The main parameters of the sequence were as follow: FOV = 30×22×22; matrix = 192×142×142; TE/TR = 3.1/6.2ms; flip angle = 20deg; reception bandwidth = 178kHz; number of excitation = 1; acquisition time = 2min3s. In order to suppress banding artifacts inherent to this sequence, the sum-of-square method was performed [55]. Thus, the total acquisition time was 8min12s.

### Numerical methods

#### Numerical methods to simulate the partial derivative equations model of spatial

The system of partial derivative equations that models the spatial metastatic growth was solved with the following numerical methods:

- a Strang splitting method for the time scheme
- a fifth order Weno finite differences scheme for the spatial resolution
- a fixed point method to solve the nonlinear equation on the pressure

#### Fitting method for the recovery of the PDE parameters

The goal of the model calibration was not to precisely determine the best parameters for describing the growth of each metastatic lesion but to obtain a range of realistic parameters to perform the study. Moreover, segmentation measurement errors, which are probably important, were not estimated. For these reasons, we used a Monte Carlo method, which was easy to implement and parallelize. Boundaries of the parameter space have been first established by an analysis of the model and biological considerations for the parameters values.

Because we did not need a very high accuracy for the fits, we did not take the shape of the metastases into account. We considered them as spherical and fitted the model on the volume dynamics only.

## Supporting Information

S1 Fig Population fit of the primary tumour dynamics. The initial volume is calibrated during the fit. Right panel: the points represent the data, the curve represents the median dynamics, and the dashed curves the percentiles. Left panel: values of the parameters resulting from the population fit of the primary tumour dynamics. NSE: normalized standard error.
(TIFF)

S2 Fig Representative MR images where size distribution of metastases could not be satisfactorily assessed. Coronal slices of three mice. Left: Day 19; Middle and right: Day 21. The metastatic foci could not be clearly segmented because the metastatic burden was very diffuse.
(TIFF)

S3 Fig Time course of the macro-metastases size distribution for different initial metastatic sizes. Top to down: Simulation of the mathematical formalism of the standard theory (i.e. dissemination and independent growth of the resulting tumour foci), using the parameter values inferred from the total metastatic burden data (total GFP signal in the lungs) using four different initial numbers of initiating metastatic cells. The results are compared to observations of macro-metastases numbers and sizes in one mouse on MRI data.
(TIFF)

S4 Fig Time course of the macro-metastases size distribution for different values of γ. The fit analysis of the GFP data was re-performed for values of *γ* ranging from 0.1 to 1, generating each time new distributions of the parameters *α*, *β* and *μ*, and simulations equivalent to Fig 3 were re-performed for the median values of parameters (inter-animal variability not shown here). Results only for *γ* = 0.1, 2/3 and 1 are shown here. Qualitatively similar results are observed concerning the size distribution metrics (in particular, number of metastases and size of the largest lesion).
(TIFF)

S5 Fig Spatial simulation of a lung metastasis. The simulated growth by the model using the fitted parameters and starting from the real shape of the observed metastasis at day 19 on the coronal MRI slice. Time course of the tumour density (up), pressure (middle), and proliferation rate fields. From left to right: day 0, day 3 and day 7. Simulations were obtained using Eqs 4–7 and the following parameter values: *γ*_0_ = 0.78 day^-1^; *Π*_0_ = 0.0026 Pa; time of simulation: T = 7 days.
(TIFF)

S6 Fig Spherical and non-spherical shapes. (A) Simulation from the segmented shape. Simulations were obtained using Eqs 4–7 and the following parameter values: *γ*_0_ = 0.78 day^-1^; *Π*_0_ = 0.0026 Pa; time of simulation: T = 7 days. (B) Simulation with the same parameters and same initial burden from a spherical shape. (C) Volume dynamics of the two simulations. The final relative difference is 2.5%.
(TIFF)

S7 Fig Evolution of the final burden with respect to the number of interacting metastases. Results of the simulation (Day 7) with different numbers of metastases: 4, 12 and 22. The parameters values are chosen among the sets of fitted parameters on individual metastatic growths. Simulations were obtained using Eqs 4–7 using the following parameter values: *γ*_0_ = 0.78 day^-1^; *Π*_0_ = 0.0026 Pa; time of simulation: T = 7 days; Initial distance between metastases: D = 0.2mm; total initial surface: S = 0.92mm^2^.
(TIFF)

S1 File Simulation movie of a lung metastasis starting from the shape of the segmented metastatic focus. The parameters have been calibrated on the volume dynamics of the metastasis. Simulations were obtained using Eqs 4–7 and the following parameter values: *γ*_0_ = 0.78 day^-1^; *Π*_0_ = 0.0026 Pa; time of simulation: T = 7 days.
(AVI)

S2 File Simulation movie of two neighbouring tumours that are growing and pushing each other by passive motion. Simulations were obtained using Eqs 4–7 and the following parameter values: *γ*_0_ = 0.78 day^-1^; *Π*_0_ = 0.0026 Pa; time of simulation: T = 7 days; initial distance between the two metastases: D = 0.2mm; initial surface of each metastasis: S = 0.46mm^2^.
(AVI)

S3 File Simulation movie of merging metastatic foci. Simulations were obtained using Eqs 4–7 and the following parameter values: *γ*_0_ = 0.78 day^-1^; *Π*_0_ = 0.0026 Pa; time of simulation: T = 7 days; number of germs = 200 in 2D.
(AVI)
