# Supplementary figures and images for: Computational Modelling of Metastasis Development in Renal Cell Carcinoma
Source: PLoS Comput Biol. 2015 Nov 23;11(11):e1004626. doi: 10.1371/journal.pcbi.1004626 (PMC4658171; doi:10.1371/journal.pcbi.1004626)

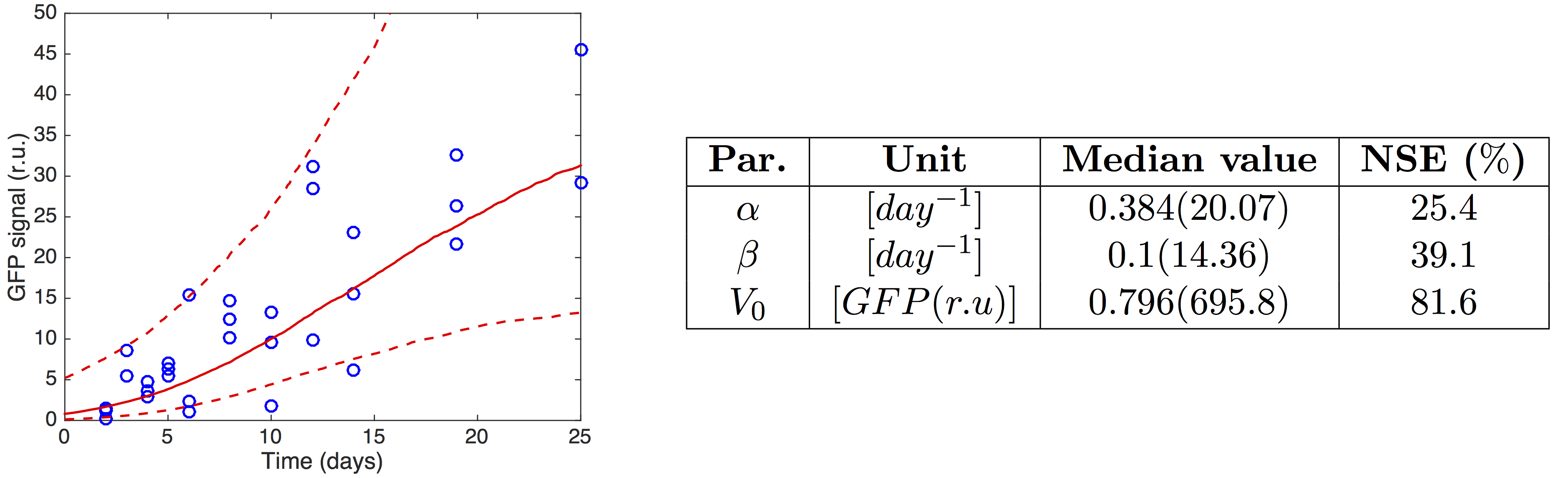

Supplement: S1 Fig — The initial volume is calibrated during the fit. Right panel: the points represent the data, the curve represents the median dynamics, and the dashed curves the percentiles. Left panel: values of the parameters resulting from the population fit of the primary tumour dynamics. NSE: normalized standard error. (TIFF) [file pcbi.1004626.s001.tiff]

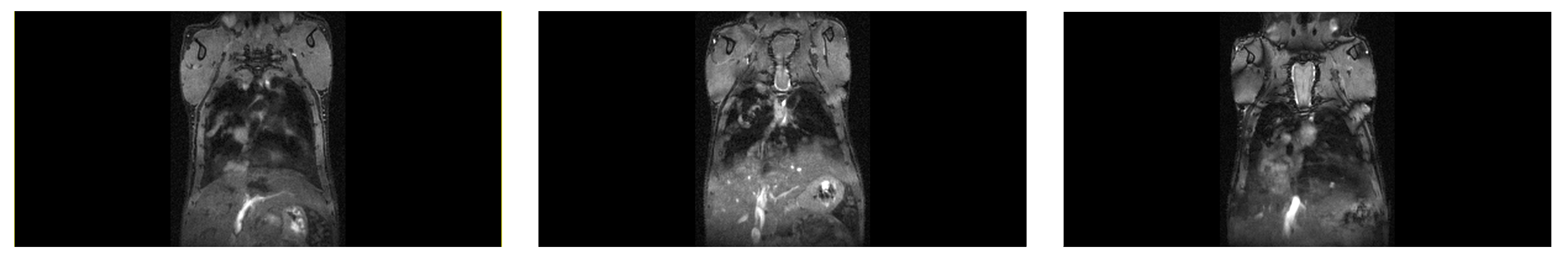

Supplement: S2 Fig — Coronal slices of three mice. Left: Day 19; Middle and right: Day 21. The metastatic foci could not be clearly segmented because the metastatic burden was very diffuse. (TIFF) [file pcbi.1004626.s002.tiff]

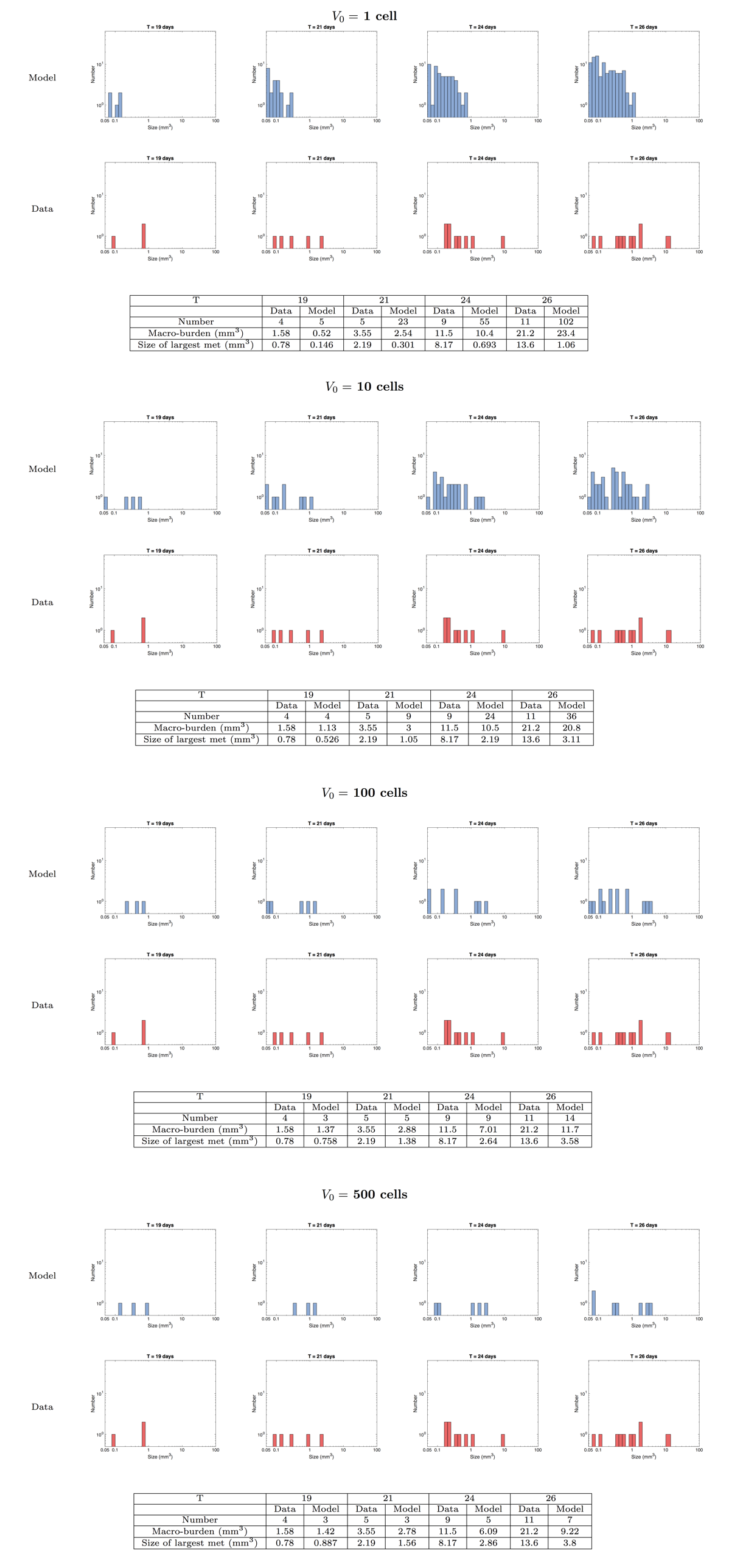

Supplement: S3 Fig — Top to down: Simulation of the mathematical formalism of the standard theory (i.e. dissemination and independent growth of the resulting tumour foci), using the parameter values inferred from the total metastatic burden data (total GFP signal in the lungs) using four different initial numbers of initiating metastatic cells. The results are compared to observations of macro-metastases numbers and sizes in one mouse on MRI data. (TIFF) [file pcbi.1004626.s003.tiff]

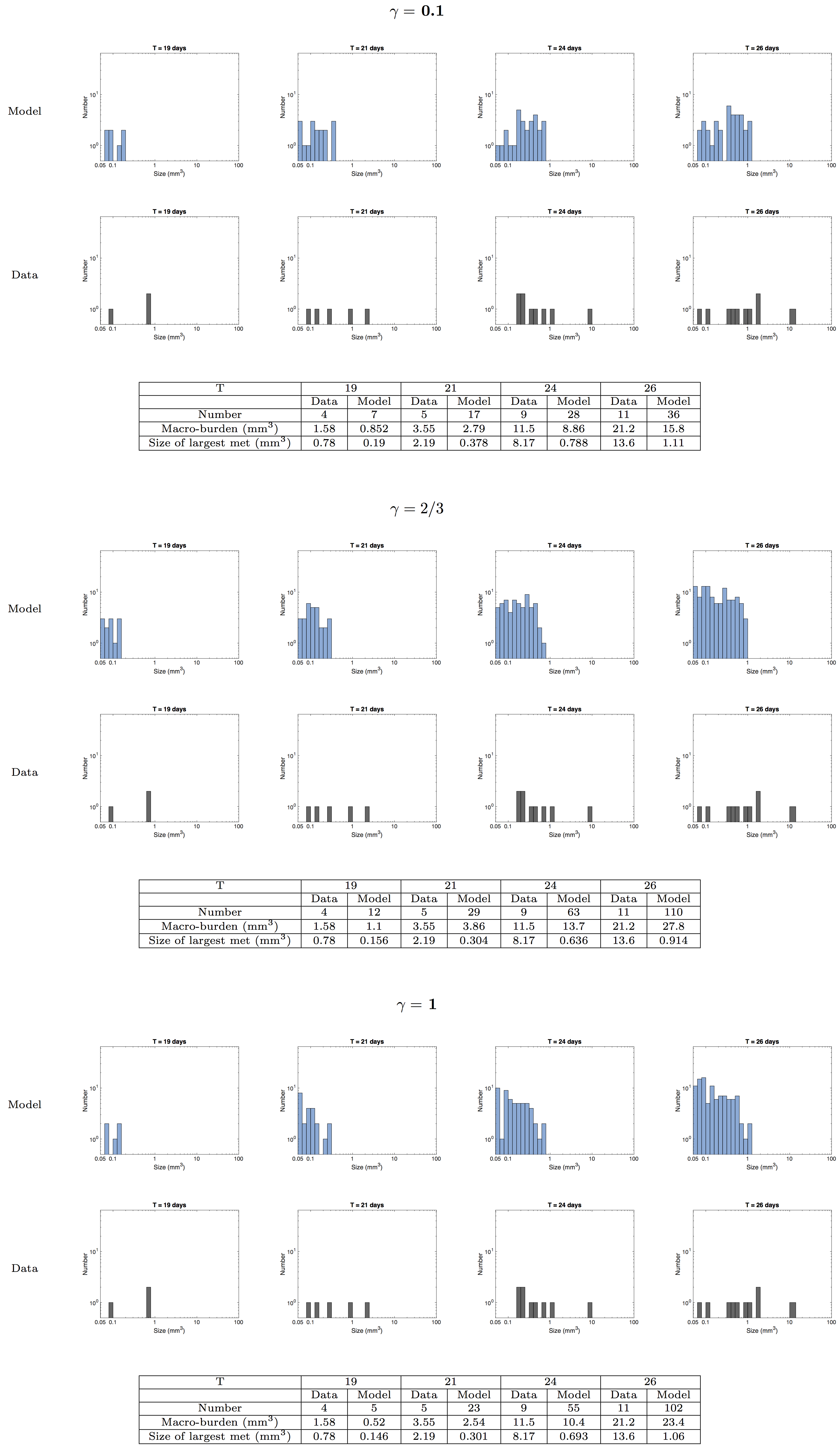

Supplement: S4 Fig — The fit analysis of the GFP data was re-performed for values of γ ranging from 0.1 to 1, generating each time new distributions of the parameters α, β and μ, and simulations equivalent to Fig 3 were re-performed for the median values of parameters (inter-animal variability not shown here). Results only for γ = 0.1, 2/3 and 1 are shown here. Qualitatively similar results are observed concerning the size distribution metrics (in particular, number of metastases and size of the largest lesion). (TIFF) [file pcbi.1004626.s004.tiff]

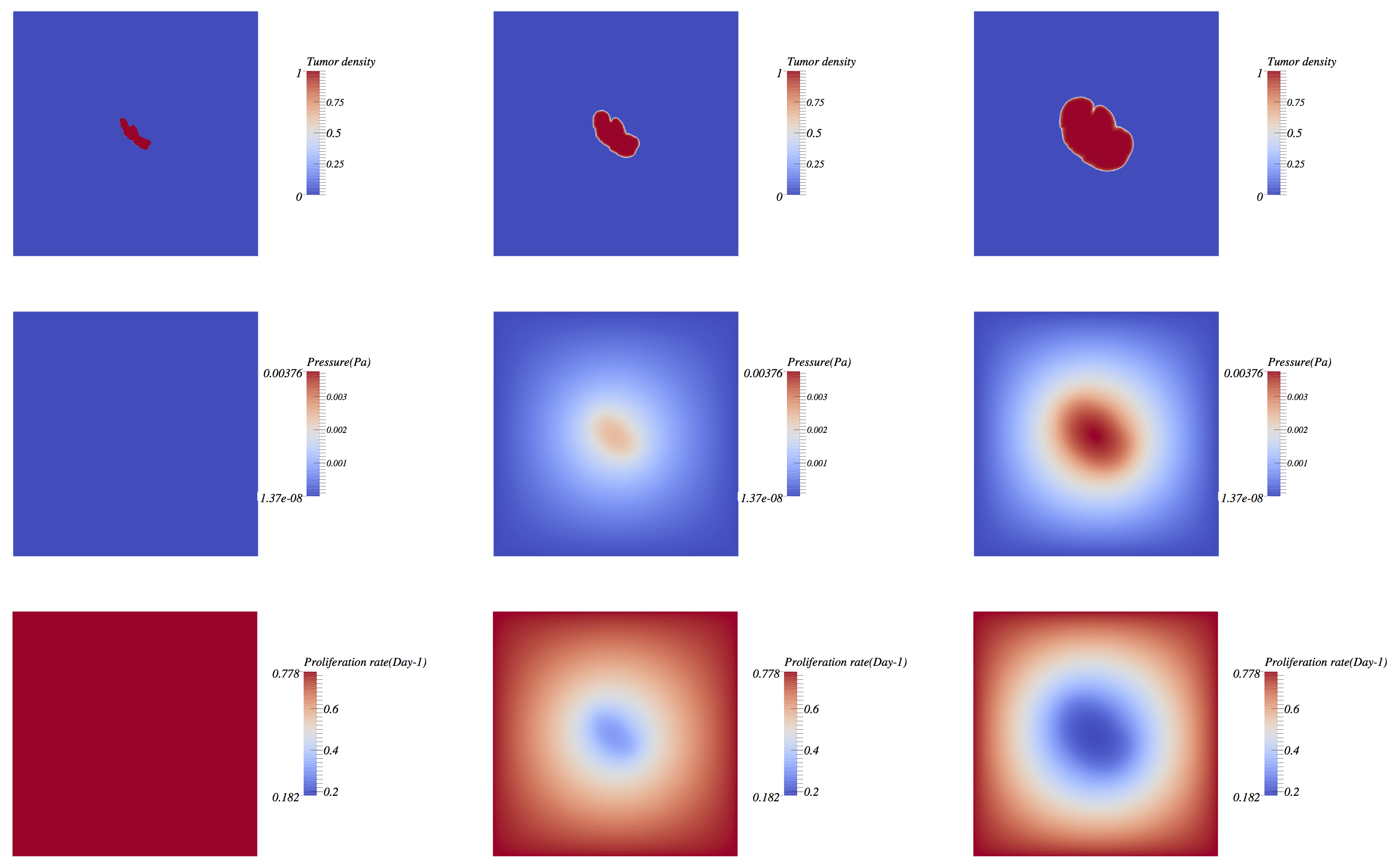

Supplement: S5 Fig — The simulated growth by the model using the fitted parameters and starting from the real shape of the observed metastasis at day 19 on the coronal MRI slice. Time course of the tumour density (up), pressure (middle), and proliferation rate fields. From left to right: day 0, day 3 and day 7. Simulations were obtained using Eqs 4–7 and the following parameter values: γ 0 = 0.78 day-1; Π 0 = 0.0026 Pa; time of simulation: T = 7 days. (TIFF) [file pcbi.1004626.s005.tiff]

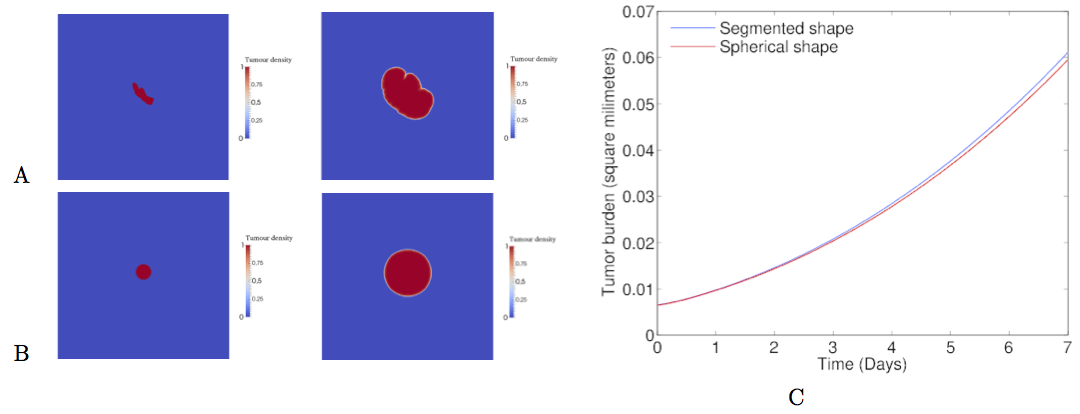

Supplement: S6 Fig — (A) Simulation from the segmented shape. Simulations were obtained using Eqs 4–7 and the following parameter values: γ 0 = 0.78 day-1; Π 0 = 0.0026 Pa; time of simulation: T = 7 days. (B) Simulation with the same parameters and same initial burden from a spherical shape. (C) Volume dynamics of the two simulations. The final relative difference is 2.5%. (TIFF) [file pcbi.1004626.s006.tiff]

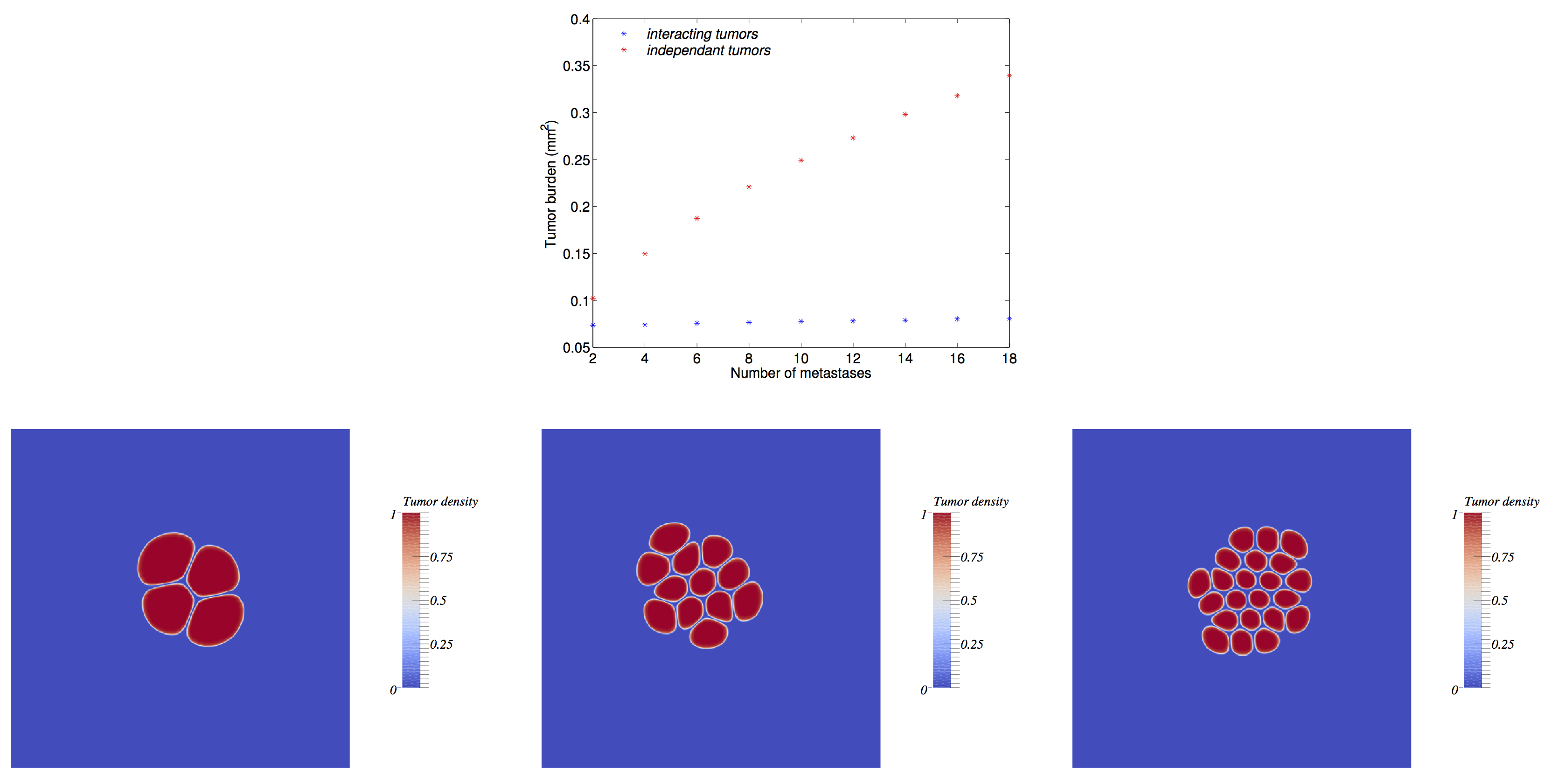

Supplement: S7 Fig — Results of the simulation (Day 7) with different numbers of metastases: 4, 12 and 22. The parameters values are chosen among the sets of fitted parameters on individual metastatic growths. Simulations were obtained using Eqs 4–7 using the following parameter values: γ 0 = 0.78 day-1; Π 0 = 0.0026 Pa; time of simulation: T = 7 days; Initial distance between metastases: D = 0.2mm; total initial surface: S = 0.92mm2. (TIFF) [file pcbi.1004626.s007.tiff]
